# The Changing Landscape of Respiratory Syncytial Virus Infections

**DOI:** 10.3390/pathogens12101196

**Published:** 2023-09-26

**Authors:** Reinout A. Bem, Job B. M. van Woensel

**Affiliations:** Pediatric Intensive Care Unit, Emma Children’s Hospital, Amsterdam University Medical Centers, Location University of Amsterdam, Meibergdreef 9, 1105 AZ Amsterdam, The Netherlands

Respiratory syncytial virus (RSV), a negative-sense, enveloped RNA virus of the family *Paramyxoviridae*, subfamily *Pneumovirinae*, and genus *Pneumovirus*, is the single most important respiratory pathogen affecting infants and young children. Since the first description of a constellate of RSV-induced clinical signs of lower respiratory tract disease (LRTD), prototypically referred to as ‘bronchiolitis’, by Hubble and Osborn in 1941 [1], the major impact on global child health of this virus became increasingly clear. While RSV-associated mortality rates have generally dropped over the last decades due to improved pediatric acute and critical supportive care [2], its associated morbidity remains high. The latest global estimates report that there are 3.6 million annual hospital admissions for RSV-LRTD in children below the age of 5 years [3]. Healthcare costs due to these hospitalizations in high-income countries are currently rising, a phenomenon largely attributable to the increased use of pediatric critical care resources [4,5]. In low-income regions without specialized facilities, around 100,000 children infected by RSV die every year [3], warning us that RSV remains among the most notable etiologies of global child mortality.

We have not yet been able to fully address the immense burden caused by RSV, either via prevention or treatment strategies [6]. Yet, several changes in this field throughout the last years announce that we may be at the advent of an important redefinition of the entire RSV landscape. In this Special Issue of Pathogens, readers can find seven diverse articles addressing several of such changes in the RSV field. All these studies make an important contribution to our insight into this respiratory pathogen.

The first change in the perspective of RSV is related to a shift in focus from acute disease to long-term sequelae. Although clinicians have long recognized that patients with RSV-LRTD can experience pulmonary dysfunction, including recurrent wheezing and asthma, in later life [7], significantly more evidence for this association has accumulated over the last two decades [8]. Importantly, the beneficial impact of preventing RSV-LRTD in preterm infants on long-term outcomes was reported by Blanken et al. in a large randomized controlled trial using the monoclonal antibody palivizumab [9]. Yet, a few years ago, via an elaborate meta-regression analysis, Brunwasser et al. challenged the theory of a causal relationship between RSV infection at a young age and the development of chronic wheezing illness [10]. In an alternative hypothesis, both RSV-LRTD and asthma may be a manifestation of an increased susceptibility to respiratory diseases from genetic and/or environmental factors. In this Special Issue of Pathogens, three papers contribute to this field on long-term outcomes. Crnković et al. report on the association of RSV-specific antibodies during infancy with the development of atopic diseases, including recurrent wheezing, in a prospective 10-year follow-up study [11]. In addition, two RSV mouse model studies zoom in on the potential mechanisms underlying the disruption of normal lung function and repair processes after acute LRTD in early life: Limkar et al. report on the protective effects of the immunobiotic agent *Lactobacillus plantarum* [12], and Lilien et al. show adverse effects on long-term pulmonary function via treatment with high-dose oxygen [13].

A second change in our perspective of RSV has come with the recognition that this pathogen is not solely important for children but is also very relevant for adult medicine. In 2005, Falsey et al. evaluated over 2500 respiratory illness cases in healthy elderly, adults with chronic heart or lung disease, and hospitalized adults, opening our eyes to a substantial disease burden caused by RSV in these populations [14]. In addition, RSV is associated with high mortality in the adult intensive care unit [15]. In this Special Issue of Pathogens, readers can find an updated overview of the most likely under-recognized incidence and impact of RSV among adults by Busack and Shorr [16].

The final and most important change for the future of RSV are current evolving clinical preventive strategies. While the treatment of RSV-LRTD remains largely limited to supportive care, the field of active and passive immunization fortunately has made significant progress in the last decade. After the first, unsuccessful attempts to develop an effective formalin-inactivated RSV vaccine in the 1960s, breakthroughs in recent years using novel vaccine development techniques have led to several promising candidates, currently approved by regulatory bodies, such as the Food and Drug Administration in the US and the European Medicines Agency of the EU. A critical step stimulating this progress was the locking of the prefusion conformation of the RSV fusion (F) glycoprotein, allowing for the exposure of its most immunogenic epitopes for vaccine development [17,18]. Agents, such as Arexvy, approved for adults over 60 years of age; Abrysvo, for adults over the age of 60 but later approved as the first maternal vaccine; and Beyfortus, a monoclonal antibody approved for passive immune protection of infants, all show promising trial results. In this Special Issue of Pathogens, Gatt et al. review both RSV therapies and the latest developments in prevention [19], and Janse et al. advocate that we should invest more in cross-utilization and collaboration between human and bovine RSV vaccine development, benefitting two separate worlds: human medicine and the veterinary industry [20].

Together, the novel vaccines may soon lead to a substantial decrease in RSV-associated disease burden, thereby redefining the future landscape of acute and chronic respiratory disease in children and adults alike. Interestingly, the recent COVID-19 pandemic taught us that the potential impact of an effective RSV prevention program may indeed be huge. At that time, the drastic reduction in close contact with humans to mitigate viral transmission resulted in a collateral disappearance of RSV-associated hospitalizations [21]. In this Special Issue of Pathogens, De Rose et al. show us an example of the effect of the COVID-19 lockdown on the incidence of RSV infections in infants in Italy, providing us with a glimpse of a brave new world without RSV [22].
